# The Hidden Cost of Age Oppression: Adultism, Ageism, and Interest in Serving Older Adults

**DOI:** 10.1093/geroni/igaa057.1996

**Published:** 2020-12-16

**Authors:** Katherine King, Kirsten Graham, Briana Reid, Molly Church, Juan Rosario

**Affiliations:** 1 William James College, Dedham, Massachusetts, United States; 2 Southern Utah University, Cedar City, Utah, United States; 3 William James College, Newton, Massachusetts, United States

## Abstract

Adultism is an underappreciated influence on young adults’ career choices and a hidden contributing factor to the geropsychology workforce shortage. This study reports on the development of an Adultist Concerns scale and its correlations with several factors relevant to careers in aging. Clinical psychology doctoral students (n = 109) completed the new scale along with measures of ageism, training interests, and experience working with older adults. The Adultist Concerns scale had strong internal consistency (α = .952) and factor loadings .853 and .929. Females scored significantly higher than males (p = .003). There were significant positive correlations between Adultist Concerns and both overall ageist behaviors (p = .002) and negative ageist behaviors specifically (p = .002). Adultist concerns were significantly negatively correlated with age (p = .000), interest in working with older adults (p = .003), and experience with this population (p = .043).

